# Spectral Camera based on Ghost Imaging via Sparsity Constraints

**DOI:** 10.1038/srep25718

**Published:** 2016-05-16

**Authors:** Zhentao Liu, Shiyu Tan, Jianrong Wu, Enrong Li, Xia Shen, Shensheng Han

**Affiliations:** 1 Key Laboratory for Quantum Optics and Center for Cold Atom Physics, Shanghai Institute of Optics and Fine Mechanics, Chinese Academy of Sciences, Shanghai 201800, China

## Abstract

The image information acquisition ability of a conventional camera is usually much lower than the Shannon Limit since it does not make use of the correlation between pixels of image data. Applying a random phase modulator to code the spectral images and combining with compressive sensing (CS) theory, a spectral camera based on true thermal light ghost imaging via sparsity constraints (GISC spectral camera) is proposed and demonstrated experimentally. GISC spectral camera can acquire the information at a rate significantly below the Nyquist rate, and the resolution of the cells in the three-dimensional (3D) spectral images data-cube can be achieved with a two-dimensional (2D) detector in a single exposure. For the first time, GISC spectral camera opens the way of approaching the Shannon Limit determined by Information Theory in optical imaging instruments.

Conventional Camera, as one of the most important appliances to get image information, records the image of an object based on the point-to-point correspondence between the object-space and the image-space. Because the correlation between pixels of image^1^ can’t be applied, the image information acquisition efficiency of such conventional point-to-point imaging mode is much lower than the Shannon Limit^2^,^3^ determined by Information Theory in optical imaging instruments^4^,^5^,^6^,^7^,^8^,^9^. Unlike the conventional direct point-to-point imaging mode, the resolution of the pixels of ghost imaging is determined by the correlation of light field fluctuations corresponding to the two pixels respectively, which can be measured on-line or pre-determined^10^,^11^. Combining with compressive sensing (CS) theory^1^,^12^,^13^,^14^,^15^,^16^, ghost imaging via sparsity constraints (GISC) has many potential applications including super-resolution imaging^17^,^18^,^19^, three-dimensional (3D) computational imaging with single-pixel detectors^20^, 3D remote sensing^21^,^22^, imaging through scattering media^23^,^24^, object tracking^25^, object authentication^26^,^27^ and X-ray Fourier transform diffraction imaging^28^,^29^,^30^.

For thermal light ghost imaging, according to the illumination source, it can be classified to two categories: ghost imaging with pseudo-thermal light and true thermal light. Ghost imaging with true thermal light and sunlight have been respectively demonstrated by detecting the temporal fluctuation of thermal light and applying the intensity correlation between the intensity distributions at the reference arm and the test arm^31^,^32^,^33^. Comparing with ghost imaging with pseudo-thermal light, this scheme of ghost imaging with true thermal light has to face the difficulty of detecting the temporal fluctuation of true thermal light which requires the response time of detector less than the coherence time of true thermal light 
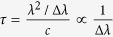
 (*λ* is the wavelength, Δ*λ* is the linewidth, *c* is the speed of light) which can be as short as femtosecond. In order to increase the coherence time of the illumining true thermal light, monochrome imaging is required which results in the vast majority of radiation energy from the target scene being filtered out, making the energy efficiency of ghost imaging applying the temporal fluctuation of true thermal light very low. Moreover, the fluctuating true thermal field needs to be split before the light field illuminating the object in the system and recorded in the reference path, which makes the scheme even more difficult to be applied in remote sensing.

In this paper, for the first time, we propose a spectral camera based on true thermal light ghost imaging via sparsity constraints (GISC spectral camera) without a splitter. GISC spectral camera modulates the true thermal light into a spatially fluctuating pseudo-thermal light using a spatial random phase modulator^34^,^35^ which, at the same time, also acts as a random grating generating the uncorrelated speckles for different wavelengths, the 3D spectral images data-cube is then modulated into a two-dimensional (2D) data plane and GISC spectral camera can achieve the whole wavelength image in a single exposure, leading to a more convenient detection process and higher energy efficiency compared to ghost imaging applying the temporal fluctuation of true thermal light. Combining with CS, GISC spectral camera can acquire the information at a rate significantly below the Nyquist rate which opens the way of approaching the Shannon Limit determined by Information Theory in optical imaging instruments^3^,^4^,^5^,^7^.

## Schematic and Resolution

**Figure 1:**
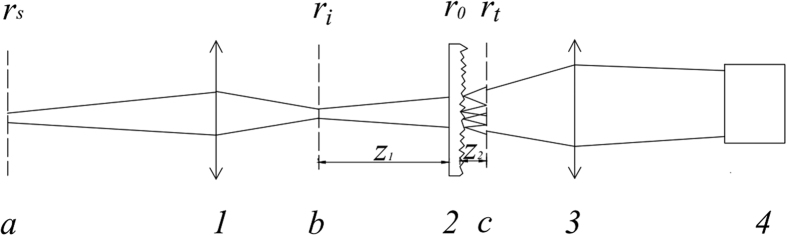
Schematic of GISC spectral camera. (**a**) The object plane; (**b**) the first image plane; (**c**) the speckles plane; (1) an imaging system; (2) a spatial random phase modulator; (3) a microscope objective; (4) CCD detector.

The schematic of GISC spectral camera is shown in Fig. 1. The system consists of (1) an imaging system, which projects the object image in the object plane ‘*a*’ onto the first image plane ‘*b*’, (2) a spatial random phase modulator, which disperses the image with different wavelengths as a random grating and modulates the image to generate the speckles in plane ‘*c*’^34^,^35^, (3) a microscope objective, which magnifies the speckles in plane ‘*c*’, and (4) a charge-coupled device (CCD) detector recording the magnified speckles.

Denoting the spectral light intensity distribution in the first image plane ‘*b*’ by *I*_*b*_(*r*_*i*_, *λ*_*l*_) and the intensity distribution in plane ‘*c*’ by *I*_*c*_(*r*_*t*_) respectively, we have^36^


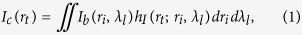


where *h*_*I*_(*r*_*t*_; *r*_*i*_, *λ*_*l*_) is the incoherent intensity impulse response function, *r*_*t*_ is the coordinate in plane ‘*c*’, *r*_*i*_ and *λ*_*l*_ are respectively the coordinate and wavelength of the light intensity distribution in the first image plane ‘*b*’. To record the pre-determined reference spatial intensity fluctuation of the pseudo-thermal light without objects, a coherent monochrome point source at pixel 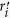 with wavelength 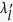 in the first image plane ‘*b*’, denoted as 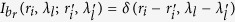, is used to illuminate the spatial random phase modulator, and the recorded light intensity 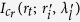 in the plane ‘*c*’ is given by


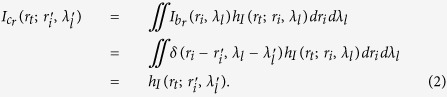


During the imaging process, the intensity distribution in the first image plane ‘*b*’ 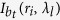 is simply the image, denoted as *T*_*i*_(*r*_*i*_, *λ*_*l*_), of the object *T*_*s*_(*r*_*s*_, *λ*_*l*_) in the object plane ‘*a*’,


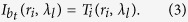


Combining Eqs (1,2) with (3), the intensity distribution 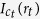 in the speckle plane ‘*c*’ is


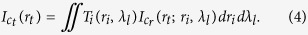


Eq. (4) shows that 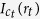 is the *T*_*i*_(*r*_*i*_, *λ*_*l*_) weighted integration of the pre-determined reference spatial intensity fluctuation of pseudo-thermal light 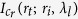. Therefore, each pixel *r*_*t*_ of CCD detector is equivalent to a measurement of the bucket detector in the test arm of ghost imaging scheme. The second-order correlation function between the spatial intensity fluctuation in the pre-determined reference arm and test arm is defined as


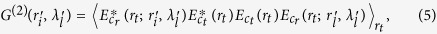


where 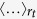 is the ensemble average about the coordinate of the light intensity distribution *r*_*t*_. Combining Eqs (2,4) with (5), the second-order correlation function 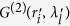 is given by


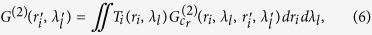


where 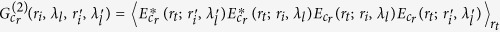 is the second-order correlation function of the light fields at different pixels and wavelengths in the first image plane ‘*b*’. In order to calculate 
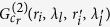
, the height autocorrelation function of the spatial random phase modulator is assumed as^37^


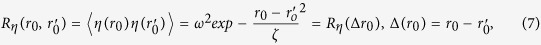


where 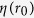 and 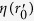 are respectively the height of the spatial random phase modulator at *r*_0_ and 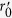, *ω* and *ζ* are respectively the height standard deviation and transverse correlation length of the spatial random phase modulator. Assuming that the light field fluctuations in the speckles plane ‘*c*’ corresponding to pixel 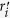 in the first image plane ‘*b*’ with wavelength 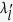 obeys the complex circular Gaussian distribution, 
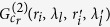
 can be written as


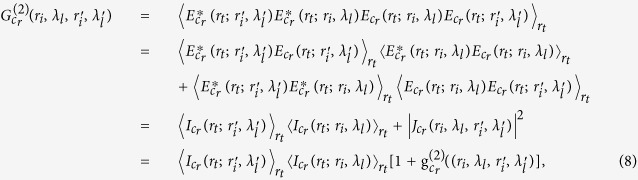


where


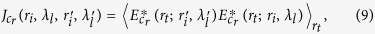



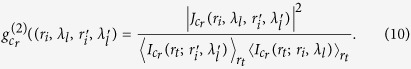



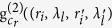
 is defined as the normalized second-order correlation function of the light fields at different pixels and wavelengths in the first image plane ‘*b*’. According to the Fresnel diffraction theorem, the light field in the speckles plane ‘*c*’ propagated from pixel 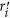 in the first image plane ‘*b*’ with wavelength 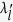 is


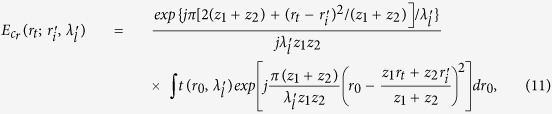


where 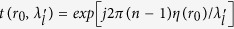 is the transmission function of the spatial random phase modulator. 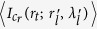 and 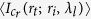 are respectively given by


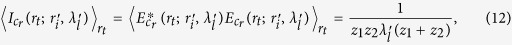



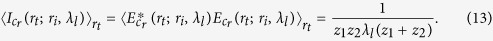


Substituting Eqs (7,9,11) into (10) yields


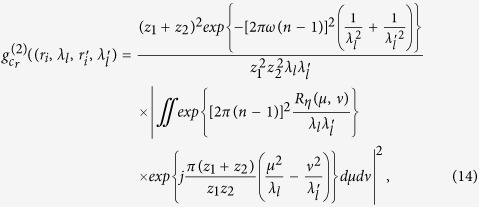


where


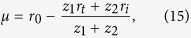



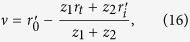



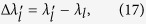



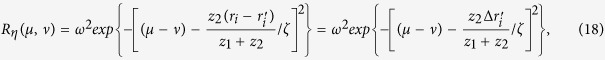



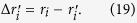


Assuming 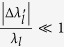, we have


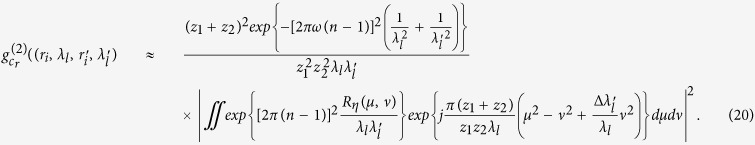


Assuming 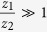, and the diameter *σ* of the illuminated region in the spatial random phase modulator by each cells of 3D data-cube in calibration satisfies *πσ*^2^/*λ*_*l*_*z*_2_ < 1, 
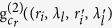
 is given by


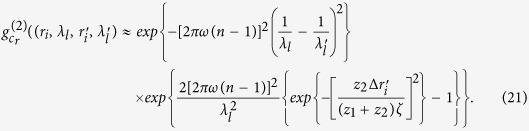


Taking Eqs (8,12,13,21) into (6), we get the correlation function of intensity fluctuations^38^


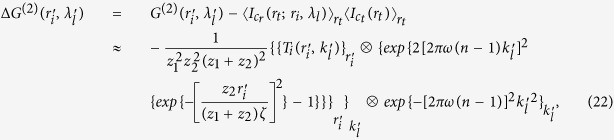


where 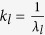, 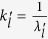, ⊗ denotes the operation of convolution. Eq. (22) specifies that 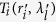 can be separated from the correlation function of intensity fluctuations 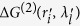, and the resolution is determined by the normalized second-order correlation 
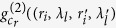
 at different pixels and wavelengths in the first image plane ‘*b*’. When 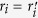, according to Eq. (14), the normalized second-order correlation function of the light fields at pixel 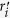 in the first image plane ‘*b*’ with two different wavelengths is given by


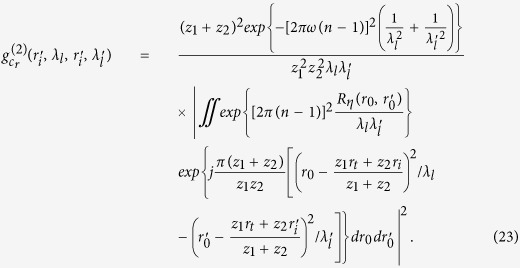


Similarly, when 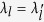, according to Eq. (14), the normalized second-order correlation function of the light fields at two different pixels in the first image plane ‘*b*’ with wavelength 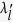 is given by


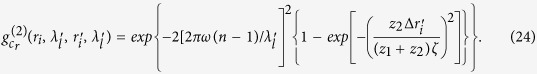


**Figure 2:**
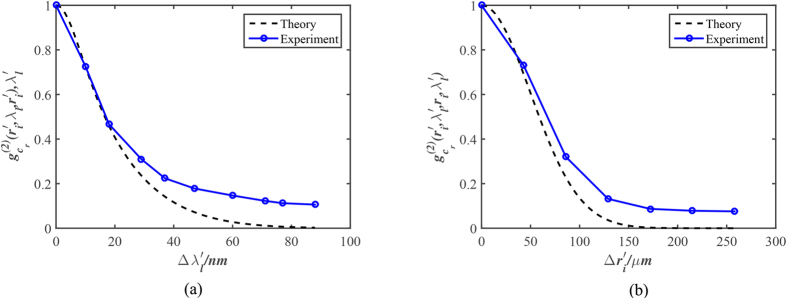
(**a**) The normalized second-order correlation function of the light fields 
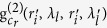
 at pixel in the first image plane ‘*b*’ with two different wavelengths; (**b**) The normalized second-order correlation function of the light fields 
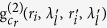
 at two different pixels in the first image plane ‘*b*’ with wavelength 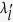.

Figure 2(a,b) respectively show the comparison of 
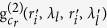
 and 
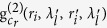
 between experiment and theory, and the experiment diagram is given in Fig. 1 with *z*_1_ = 20 *mm*, *z*_2_ = 0.3 *mm*, *ω* = 2.1 *μm*, *ζ* = 16.75 *μm*, *n* = 1.516 and the central wavelength 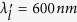.

## The Measurement Matrix & Reconstruction Algorithm

There are many methods to improve the imaging quality of ghost imaging^39^,^40^,^41^. However, ghost imaging reconstructions based on the ensemble statistics cannot provide the criterion of the necessary number of sampling for a perfect imaging, which makes it impossible to optimize the design of ghost imaging system. Combining with CS which provides the recovery condition of perfect reconstruction, the quantitative analysis for the necessary measurements data can be made. Under the framework of CS theory, the measurement matrix of GISC spectral camera is obtained as follows: each of the speckle intensity distributions generated by a point light source at pixel 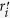 in the 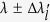 spectrum band in the first image plane ‘*b*’ is recorded by the randomly selected 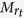 pixels of CCD detector and reshaped as a column vector of length *M* of the measurement matrix. Repeating the process for all the *N* image pixels in the first image plane ‘*b*’ and all the *L* spectral bands, one may have the pre-determined random measurement matrix *A*_*M*×*K*_, where *K* = *L* × *N*. If we denote the unknown spectral object image as a *K*-dimensional column vector *X*_*K*×1_, and reshape the modulated object intensity distribution recorded by the same *M* pixels of CCD detector in a similar way as a column vector *Y*_*M*×1_, then we may have the discrete from Eq. (4),


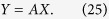


Spectral object image is usually both spatially and spectrally correlated, which has already been utilized in spectral image reconstructions^42^,^43^,^44^. The reconstruction of the spectral object image can generally be regarded as solving a minimization problem which penalizes both the *l*_1_ norm and the nuclear norm of the data matrix:


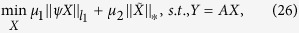


where 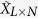 a matrix representation of the spectral object image whose columns represent different bands of the spectral object image, *ψ* the sparsifying transform, *μ*_1_ and *μ*_2_ the weight coefficients and *μ*_1_, *μ*_2_ > 0. In this work, we use a modified approach based on the method described by Eq. (26)^45^:


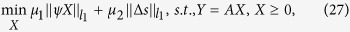


where 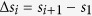, is the subtraction of the largest singular value *s*_1_ and the other *s*_*i*_. The solution of Eq. (27) tends to have a simultaneous low-effective-rank and sparse structure, which much improves the reconstruction quality with low sampling rate.

## Experimental Results

**Figure 3:**
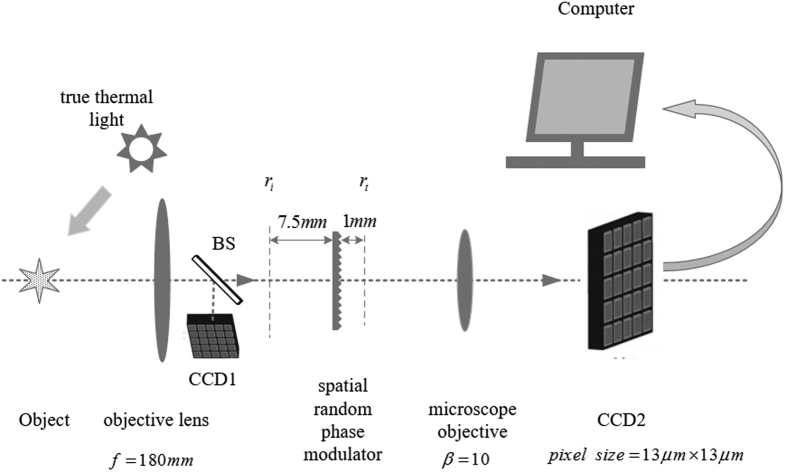
Experimental setup of GISC spectral camera.

In the experimental setup of GISC spectral camera shown in Fig. 3, the imaging system (Tamron AF70-300 *mm* f/4-5.6) with focal length of *f* = 180 *mm* projects the object image onto the first image plane, a beam splitter (BS) with split ratio 50:50 splits the light field into two paths, CCD1 detector (AVT Sting F-504C with pixel size of 3.45 *μm* × 3.45 *μm*) is placed in one of the two paths at the position of the first image plane of the system to obtain the conventional image of the object for comparison, a spatial random phase modulator (SIGMA KOKI CO., LTD. DFSQ1-30C02-1000) disperses the images with different wavelengths acting as a random grating and modulates the image to generate the speckles, a microscope objective with magnification *β* = 10 and the numerical aperture *N*.*A*. = 0.25 magnifies the speckles which are then recorded by CCD2 detector (Andor iKon-M) with the pixel size 13 *μm* × 13 *μm*. The first image plane is divided into *N*_*x*_ × *N*_*y*_ = 140 × 140 pixels with the square of each pixel approximately equal to Δ*r*_*s*_ determined by the Eq. (24). The number of spectrum bands for single exposure is 7, and the images in two wavelength ranges of 520 ~ 580 *nm* and 620 ~ 680 *nm* are respectively obtained in two exposures, while the theoretical spectral resolution is 20 *nm* in the experimental setup according to Eq. (23).

**Figure 4:**
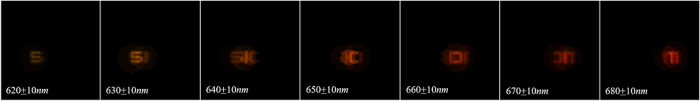
The original spectral images of ‘SIOM’ obtained by CCD1 detector placed on the first image plane ‘*b*’ with corresponding narrowband filter in front of it, showing all the channels from 620 ~ 680 *nm*.

**Figure 5:**
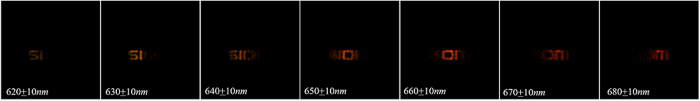
The reconstructed spectral images of ‘SIOM’ with 30% sampling rate of 3D date-cube, showing all the channels from 620 ~ 680 *nm*.

In order to compare the spectral & spatial resolution of GISC spectral camera with the theoretical resolution, as shown in Fig. 4, the spectral object ‘SIOM’ with different parts passing through different wavelengths has been selected, and the illuminating source is a xenon lamp. The original spectral images of ‘SIOM’ obtained by CCD1 detector placed in the first image plane ‘*b*’ with corresponding narrowband filter in front of it are shown in Fig. 4 (pixel size is equal to the theoretical resolution of reconstructed images by GISC spectral camera for comparing them). The corresponding modulated object intensity distribution *Y* is achieved by CCD2 detector of GISC spectral camera and the reconstructed spectral images of ‘SIOM’ with 30% sampling rate of 3D date-cube are shown in Fig. 5. The comparison between the original and reconstructed spectral images shows that the resolution of GISC spectral camera is in accordance with the theoretical calculation.

**Figure 6:**
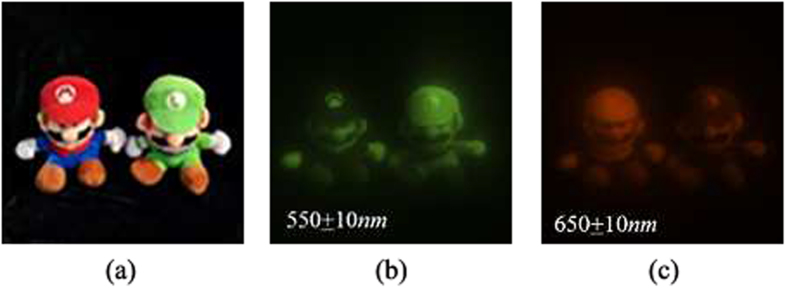
Mario & Luigi taken by (**a**) a camera; (**b**) CCD1 detector passing through narrowband filters of 550 ± 10 *nm*; (**c**) CCD1 detector passing through narrowband filters of 650 ± 10 *nm*.

**Figure 7:**
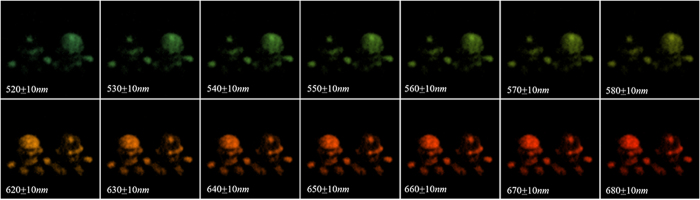
The reconstructed spectral images of Mario & Luigi with 30% sampling rate of 3D date-cube, showing all the channels from 520 ~ 580 *nm* and 620 ~ 680 *nm*.

The images of the outdoor scene consisting of Mario & Luigi with sunlight illumination are shown in Fig. 6. Figure 6(a) is obtained by a camera, while Fig. 6(b,c) respectively show the pictures taken by CCD1 detector with narrowband filters of 550 ± 10 *nm* and 650 ± 10 *nm* in front of it (pixel size is equal to the theoretical resolution of reconstructed images by GISC spectral camera for the sake of comparison). The reconstructed spectral images of Mario & Luigi with 30% sampling rate of 3D date-cube are shown in Fig. 7. The experimental results show that the spectral imaging ability of GISC spectral camera for complex scenes is also pretty good.

## Discussion and Conclusion

Based on Information Theory, the transmitted information of an imaging system can be described by the entropy^2^,^3^


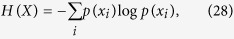


where *p*(*x*_*i*_) is the probability of *x*_*i*_ occurrence. For the conventional direct point-of-object-space to point-of-image-space imaging mode, the conditional entropy *H*(*X*|*Y*) = 0, and thus the channel capacity of the conventional monochrome camera is


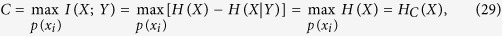


where *I*(*X*; *Y*) is the mutual information, 
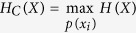
 the maximum information entropy of source *X* for conventional imaging instrument, which is the Shannon Limit of the imaging system. According to the principle of maximum entropy^1^, the information content of an image is maximized when *p*(*x*_*i*_) is Gaussian distribution with average power constraints, which doesn’t contain any useful information. Therefore, the entropy of the image with structured information *H*(*X*) has


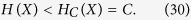


Eq. (30) shows that the image information acquisition efficiency of such conventional point-to-point imaging mode is lower than the Shannon Limit determined by Information Theory in optical imaging instruments. The channel capacity of an imaging system based on Information Theory for conventional optical imaging instruments is^4^,^5^,^6^,^7^


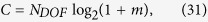


where *m* is signal to noise ratio (SNR), *N*_*DOF*_ is degrees of freedom and has


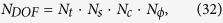


where *N*_*t*_, *N*_*s*_, *N*_*c*_ and *N*_*ϕ*_ are respectively time, spatial, color and polarization degrees of freedom. Spatial degrees of freedom *N*_*s*_ has^6^


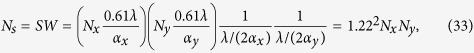


where *S* is the image area, *W* is the space bandwidth, *α*_*x*_, *α*_*y*_ and *N*_*x*_, *N*_*y*_ are respectively the image-space aperture angle and the resolved pixel number in the image-space of coordinate *x* and *y*. The color degrees of freedom *N*_*c*_ depend on the number of spectral channels, while polarization degrees of freedom *N*_*ϕ*_ is determined by the independent polarization state. According to Eqs (28,31,33), the channel capacity of the con-ventional camera in our experiment (where 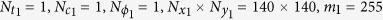) is 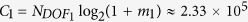, and the corresponding transmitted information of Fig. 6(b) is 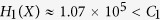. In order to transmit the 520 ~ 580 *nm* wavelength ranges data, the required channel capacity of the conventional camera (where 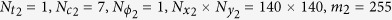) is *C* ≈ 1.63 × 10^6^, while the required channel capacity in GISC spectral camera with 30% sampling rate in our experiment is *C*_3_ ≈ 4.90 × 10^5^. *C*_3_ < *C*_2_ shows that GISC spectral camera has the higher information acquisition efficiency in a single exposure compared to the conventional camera. With the development of optical imaging technology, many new imaging technologies (such as CT image^46^) are not based on the point-to-point imaging mode. However, because the correlation between pixels of image data doesn’t be applied in the imaging reconstruction algorithm, the information acquisition efficiency of those new coding imaging technology also can’t approaching the Shannon Limit determined by Information Theory for conventional optical imaging instruments. However, GISC imaging solution applies a spatial random phase modulation to satisfy the restricted isometry property (RIP)^17^ required by applying CS that makes the improvement of information acquisition efficiency of the imaging system possible. Comparing with CS imaging technology (such as Single-Pixel Imaging via Compressive Sampling^47^, coded aperture snapshot spectral imagers^48^), which forces on the compressive sampling of electric signal after photoelectric conversion to improve the channel capacity utilization efficiency of the electric signal, GISC imaging solution improves the optical channel capacity utilization efficiency and achieves the compressive sampling of the image data during the imaging acquisition process, which opens the way of approaching the Shannon Limit determined by Information Theory in optical imaging instruments. As a new optical imaging technology, GISC spectral camera provides a unique solution for the spectral imaging of dynamic processes. This GISC imaging solution may also be expanded to other multi-dimensional information (such as polarization information) acquisition^49^, ultra-fast measurement^50^, and super-resolution imaging^18^,^51^,^52^.

## Additional Information

**How to cite this article**: Liu, Z. *et al*. Spectral Camera based on Ghost Imaging via Sparsity Constraints. *Sci. Rep*. **6**, 25718; doi: 10.1038/srep25718 (2016).

## References

[b1] Jacobs E., Fisher Y. & Boss R. Image compression: A study of the iterated transform method. Signal Process. 29, 251–263 (1992).

[b2] SHANNON C. A mathematical theory of communication. Bell Sys. Tech. Jour. 27, 397–423, 623–656 (1948).

[b3] Cover T. M. & Thomas J. A. Elements of information theory 657–687 (John Wiley & Sons, 2012).

[b4] Elias P. Optics and communication theory. JOSA 43, 229–232 (1953).

[b5] Francia G. Resolving power and information. JOSA 45, 497–499 (1955).

[b6] Di Francia G. T. Degrees of freedom of an image. JOSA 59, 799–804 (1969). 10.1364/josa.59.000799 5802951

[b7] Tan W. Optical information theory-retrospect and prospect. Optics Precis. Eng. 3, 17–22 (1982).

[b8] Huck F. O., Fales C. L., Alter-Gartenberg R., Park S. K. & Rahman Z.-u. Information-theoretic assessment of sampled imaging systems. Opt. Eng. 38, 742–762 (1999).

[b9] Strange B. A., Duggins A., Penny W., Dolan R. J. & Friston K. J. Information theory, novelty and hippocampal responses: unpredicted or unpredictable? Neural Netw. 18, 225–230 (2005). 15896570 10.1016/j.neunet.2004.12.004

[b10] Kolobov M. I. Quantum imaging 79–110 (Springer Science & Business Media, 2007).

[b11] Shapiro J. H. & Boyd R. W. The physics of ghost imaging. Quantum Inf. Process. 11, 949–993 (2012).

[b12] Gonzalez R. C., Woods R. E. & Eddins S. L. Digital image processing using MATLAB (Pearson Education India, 2004).

[b13] Donoho D. L. Compressed sensing. IEEE Trans. Inform. Theory 52, 1289–1306 (2006).

[b14] Candès E. J., Romberg J. & Tao T. Robust uncertainty principles: Exact signal reconstruction from highly incomplete frequency information. IEEE Trans. Inform. Theory 52, 489–509 (2006).

[b15] Eldar Y. C. & Kutyniok G. Compressed sensing: theory and applications (Cambridge University Press, 2012).

[b16] Wu J. . Snapshot compressive imaging by phase modulation. Acta Phys. Sin-CH ED 34, 1011005 (2014).

[b17] Gong W. & Han S. Experimental investigation of the quality of lensless super-resolution ghost imaging via sparsity constraints. Phys. Lett. A 376, 1519–1522 (2012).

[b18] Wang H., Han S. & Kolobov M. I. Quantum limits of super-resolution of optical sparse objects via sparsity constraint. Opt. Express 20, 23235–23252 (2012). 23188288 10.1364/OE.20.023235

[b19] Gong W. & Han S. High-resolution far-field ghost imaging via sparsity constraint. Sci. Rep. 5, 9280 (2015). 25787897 10.1038/srep09280 PMC4365410

[b20] Sun B. . 3d computational imaging with single-pixel detectors. Science 340, 844–847 (2013). 23687044 10.1126/science.1234454

[b21] Zhao C. . Ghost imaging lidar via sparsity constraints. Appl. Phys. Lett. 101, 141123 (2012).

[b22] Gong W. . Three-dimensional ghost imaging ladar. *arXiv preprint arXiv:1301.5767* (2013).

[b23] Gong W. & Han S. Correlated imaging in scattering media. Opt. Lett. 36, 394–396 (2011). 21283201 10.1364/OL.36.000394

[b24] Bina M. . Backscattering differential ghost imaging in turbid media. Phys. Rev. Lett. 110, 083901 (2013). 23473147 10.1103/PhysRevLett.110.083901

[b25] Magaña-Loaiza O. S., Howland G. A., Malik M., Howell J. C. & Boyd R. W. Compressive object tracking using entangled photons. Appl. Phys. Lett. 102, 231104 (2013).

[b26] Chen W. & Chen X. Object authentication in computational ghost imaging with the realizations less than 5% of nyquist limit. Opt. Lett. 38, 546–548 (2013). 23455131 10.1364/OL.38.000546

[b27] Xu X., Li E., Yu H., Gong W. & Han S. Morphology separation in ghost imaging via sparsity constraint. Opt. Express 22, 14375–14381 (2014). 24977534 10.1364/OE.22.014375

[b28] Cheng J. & Han S. Incoherent coincidence imaging and its applicability in x-ray diffraction. Phys. Rev. Lett. 92, 093903 (2004). 15089466 10.1103/PhysRevLett.92.093903

[b29] Zhang M. . Lensless fourier-transform ghost imaging with classical incoherent light. Phys. Rev. A 75, 021803 (2007).

[b30] Wang H. & Han S. Coherent ghost imaging based on sparsity constraint without phase-sensitive detection. Europhys. Lett. 98, 24003 (2012).

[b31] Zhang D., Zhai Y.-H., Wu L.-A. & Chen X.-H. Correlated two-photon imaging with true thermal light. Opt. Lett. 30, 2354–2356 (2005). 16196317 10.1364/ol.30.002354

[b32] D’Angelo M. & Shih Y. Quantum imaging. Laser Phys. Lett. 2, 567–596 (2005).

[b33] Liu X.-F. . Lensless ghost imaging with sunlight. Opt. Lett. 39, 2314–2317 (2014). 24978981 10.1364/OL.39.002314

[b34] Giglio M., Carpineti M. & Vailati A. Space intensity correlations in the near field of the scattered light: a direct measurement of the density correlation function g (r). Phys. Rev. Lett. 85, 1416 (2000). 10970518 10.1103/PhysRevLett.85.1416

[b35] Cerbino R. . X-ray-scattering information obtained from near-field speckle. Nat. Phys. 4, 238–243 (2008).

[b36] Goodman J. W. Introduction to Fourier optics 154–160 (Roberts and Company Publishers, 2005).

[b37] Cheng C.-F., Qi D.-P., Liu D.-L. & Teng S.-Y. The computational simulations of the gaussian correlation random surface and its light-scattering speckle field and the analysis of the intensity probability density. Acta Phys. Sin-CH ED 48, 1635–1643 (1999).

[b38] Gatti A., Brambilla E., Bache M. & Lugiato L. A. Ghost imaging with thermal light: comparing entanglement and classicalcorrelation. Phys. Rev. Lett. 93, 093602 (2004). 15447100 10.1103/PhysRevLett.93.093602

[b39] Chan K. W. C., O’Sullivan M. N. & Boyd R. W. High-order thermal ghost imaging. Opt. Lett. 34, 3343–3345 (2009). 19881588 10.1364/OL.34.003343

[b40] Gong W. & Han S. A method to improve the visibility of ghost images obtained by thermal light. Phys. Lett. A 374, 1005–1008 (2010).

[b41] Ferri F., Magatti D., Lugiato L. & Gatti A. Differential ghost imaging. Phys. Rev. Lett. 104, 253603 (2010). 20867377 10.1103/PhysRevLett.104.253603

[b42] Oymak S., Jalali A., Fazel M., Eldar Y. C. & Hassibi B. Simultaneously structured models with application to sparse and low-rank matrices. IEEE Trans. Inform. Theory 61, 2886–2908 (2015).

[b43] Golbabaee M. & Vandergheynst P. Compressed sensing of simultaneous low-rank and joint-sparse matrices. *arXiv preprint arXiv:1211.5058* (2012).

[b44] Golbabaee M. & Vandergheynst P. Joint trace/tv norm minimization: A new efficient approach for spectral compressive imaging. In *Image Processing (ICIP), 2012 19th IEEE International Conference on*, 933–936 (IEEE, 2012).

[b45] Zhang H., He W., Zhang L., Shen H. & Yuan Q. Hyperspectral image restoration using low-rank matrix recovery. IEEE Trans. Ceosci. Remote Sens. 52, 4729–4743 (2014).

[b46] Hsieh J. Computed tomography: principles, design, artifacts, and recent advances (SPIE: Bellingham, WA, , 2009).

[b47] Duarte M. F. . Single-pixel imaging via compressive sampling. IEEE Signal Process. Mag. 25, 83 (2008).

[b48] Kittle D., Choi K., Wagadarikar A. & Brady D. J. Multiframe image estimation for coded aperture snapshot spectral imagers. Appl. Optics 49, 6824–6833 (2010). 10.1364/AO.49.006824 21173812

[b49] Morgan S. P. & Stockford I. Surface-reflection elimination in polarization imaging of superficial tissue. Opt. Lett. 28, 114–116 (2003). 12656502 10.1364/ol.28.000114

[b50] Gao L., Liang J., Li C. & Wang L. V. Single-shot compressed ultrafast photography at one hundred billion frames per second. Nature 516, 74–77 (2014). 25471883 10.1038/nature14005 PMC4270006

[b51] Donoho D. L. Superresolution via sparsity constraints. SIAM J. Math. Anal. 23, 1309–1331 (1992).

[b52] Candès E. J. & Fernandez-Granda C. Towards a mathematical theory of super-resolution. Commun. Pur. Appl. Math. 67, 906–956 (2014).

